# Post-concussive symptoms mediate the relationship between sleep problems and participation restrictions among veterans with mild traumatic brain injury

**DOI:** 10.3389/fresc.2022.964420

**Published:** 2022-10-12

**Authors:** Adam R. Kinney, Xiang-Dong Yan, Alexandra L. Schneider, Samuel King, Jeri E. Forster, Nazanin Bahraini, Lisa A. Brenner

**Affiliations:** ^1^ Department of Veterans Affairs (VA) Rocky Mountain Mental Illness Research Education and Clinical Center (MIRECC) for Veteran Suicide Prevention, Aurora, CO, United States; ^2^ Department of Physical Medicine and Rehabilitation, School of Medicine, University of Colorado Anschutz Medical Campus, Aurora, CO, United States; ^3^ Departments of Physical Medicine and Rehabilitation and Psychiatry, School of Medicine, University of Colorado Anschutz Medical Campus, Aurora, CO, United States; ^4^ Departments of Physical Medicine and Rehabilitation, Psychiatry, and Neurology, School of Medicine, University of Colorado Anschutz Medical Campus, Aurora, CO, United States

**Keywords:** concussion, veterans, sleep, participation, postconcussive symptoms, neurobehavioral symptoms, mild traumatic brain injury (mTBI)

## Abstract

**Background:**

Sleep problems are common among Veterans with mild traumatic brain injury (mTBI) and may contribute to participation restrictions. However, explanatory mechanisms underlying this relationship are poorly understood. Sleep problems are associated with post-concussive symptoms (e.g., headaches). In turn, post-concussive symptoms contribute to participation restrictions. We hypothesized that post-concussive symptom severity mediates the purported relationship between sleep problems and participation restrictions among Veterans with mTBI.

**Materials and Methods:**

This study was a retrospective analysis of clinical data among 8,733 Veterans with mTBI receiving Veterans Health Administration outpatient care. Sleep problems (yes/no) were identified using the sleep-related item from the Neurobehavioral Symptom Inventory (NSI). Post-concussive symptoms were measured using remaining NSI items. Participation restrictions were measured using the Mayo-Portland Adaptability Inventory Participation Index. We specified a latent variable path model to estimate relationships between: (1) sleep problems and three latent indicators of post-concussive symptoms [vestibular-sensory (e.g., headache)]; mood-behavioral [e.g., anxiety]; cognitive [e.g., forgetfulness]); and, (2) the three latent indicators of post-concussive symptoms and two latent indicators of participation restrictions (social and community participation [e.g., leisure activities]; productivity [e.g., financial management]). We examined the indirect effects of sleep problems upon participation restrictions, as mediated by post-concussive symptoms. Estimates were adjusted for sociodemographic factors (e.g., age), injury characteristics (e.g., blast), and co-morbid conditions (e.g., depression).

**Results:**

87% of Veterans reported sleep problems. Sleep problems were associated with greater social and community participation restrictions, as mediated by mood-behavioral (*β* = 0.41, *p* < 0.001) and cognitive symptoms (*β* = 0.13, *p* < 0.001). There was no evidence that vestibular-sensory symptoms mediated this relationship (*β* = -0.01, *p* = 0.48). Sleep problems were associated with greater productivity restrictions, as mediated by vestibular-sensory (*β* = 0.16, *p* < 0.001) and cognitive symptoms (*β* = 0.14, *p* < 0.001). There was no evidence that mood-behavioral symptoms mediated this relationship (*β* = 0.02, *p* = 0.37).

**Discussion:**

Findings suggest that evidence-based sleep treatment should occupy a prominent role in the rehabilitation of Veterans with mTBI. Indirect effects of sleep problems differed when considering impact on social and community participation vs. productivity, informing individualized rehabilitative care for Veterans with mTBI.

## Introduction

Participation has been defined as involvement in activities which facilitate the fulfillment of socially defined roles (e.g., parent), connection with others, and the elicitation of subjective meanings (e.g., perceived competence) (1). Veterans of Operations Enduring Freedom, Iraqi Freedom, and New Dawn (post-9/11 Veterans) are at substantial risk for mild traumatic brain injury (mTBI) (2–5), which may contribute to participation restrictions (6). Participation restrictions among those with mTBI are in part due to post-concussive symptoms, or neurobehavioral symptoms that arise following the injury, which span across vestibular (e.g., balance), sensory (e.g., headache), behavioral (e.g., irritability), and/or cognitive (e.g., forgetfulness) domains (7–9). While sequalae of mTBI typically resolve within three months of the injury event, a notable subset experience persistent symptoms (10–15), posing ongoing risk for participation challenges among Veterans with mTBI.

Sleep problems are among the most prevalent and disabling post-concussive symptoms among post-9/11 Veterans. A nationwide study of post-9/11 Veterans revealed that 85% of those with clinician-confirmed traumatic brain injury (TBI; i.e., mild, moderate, or severe) experience sleep disturbance (16). Individuals with mTBI experience multi-faceted sleep problems, including greater daytime sleepiness, lesser total sleep time and difficulties with initiating and maintaining sleep (17). Poor sleep quality may prolong recovery from mTBI and exacerbate risk for participation restrictions (18–20). For example, sleep disturbances have been shown to predict decreased function up to a year following a mTBI, even after adjusting for other relevant factors (e.g., psychological distress) (21). However, the unique effect of sleep problems on participation restrictions among Veterans with mTBI is not well understood. Further, there is a need to establish empirical support for explanatory mechanisms underlying the purported relationship between participation restrictions and post-concussive sleep problems.

Post-concussive symptoms may serve as a mediating factor explaining the relationship between sleep problems and participation restrictions among Veterans with mTBI. The link between poor sleep quality and neurophysiological dysfunction is well-documented (22, 23). Likewise, sleep disruption may undermine these same neurophysiological processes and impede post-concussive recovery (18, 20). Indeed, poor sleep quality is linked to more severe post-concussive symptoms across vestibular, sensory, behavioral, and cognitive domains (24, 25). In turn, such post-concussive symptoms are a robust risk factor for participation restrictions among post-9/11 Veterans with TBI (8, 9).

The purpose of this study was to examine whether post-concussive symptoms mediate the relationship between sleep problems and participation restrictions among post-9/11 Veterans with mTBI. There is theoretical and empirical support for relationships between: (1) sleep problems and post-concussive symptoms (24, 25); and, (2) post-concussive symptoms and participation restrictions (8, 9). Thus, we hypothesized that the relationship between sleep problems and participation restrictions would be mediated by post-concussive symptoms. See supplementary material for a conceptual model illustrating hypothesized relationships. Understanding potential mechanisms (i.e., post-concussive symptoms) by which sleep problems and participation restrictions are related may inform strategies to enhance rehabilitative care for Veterans with mTBI.

## Materials and methods

### Participants and procedures

This study was a retrospective analysis of clinical data among a national sample of Veterans with mTBI who received outpatient care in the Veterans Health Administration (VHA) between 2012 and 2020. Study procedures were approved by the local Institutional Review Board and VA committees. Data was extracted from the Comprehensive TBI Evaluation (CTBIE) database, the National Veterans TBI Health Registry database (26), and the Corporate Data Warehouse, which stores VHA electronic medical record data (e.g., diagnoses). The CTBIE is an extensive clinical interview conducted with Veterans who screen positive for a potential TBI, per the VHA TBI screening protocol (27). The interview includes a thorough examination of the injury event (e.g., severity and mechanism) as well as severity of specific post-concussive symptoms. Starting in 2012, the administration of the Mayo-Portland Adaptability Inventory Participation Index (M2PI) subscale within 30 days of the CTBIE was encouraged (see details below) (8, 28). M2PI results are uploaded to the National Veterans TBI Health Registry database (26).

Veterans were included in this study if the following was documented: (1) a complete CTBIE; (2) a clinician-confirmed mTBI, per criteria specified in the VA/DoD Clinical Practice Guideline for mTBI (e.g., loss of consciousness: 0–30 min) (29); and, (3) a complete self-reported M2PI record within 30 days of the CTBIE. Records were excluded for Veterans who had: (1) an inpatient stay which overlapped with the date at which the CTBIE and/or M2PI was administered; and, (2) an M2PI administered by a clinician or significant other, as prior research indicates discrepancies across reporting method (30). For individuals with more than one CTBIE and/or M2PI, the first chronological record was used. We excluded six eligible individuals with incomplete data on covariates. The eligible study cohort for this study included 8,733 Veterans with clinician-confirmed mTBI.

### Measures

#### Sleep problems

Presence of *sleep problems* (yes vs. no) was determined using the corresponding item from the Neurobehavioral Symptom Inventory (NSI), a 22-item assessment of post-concussive symptoms (31). Participants rated their “difficulty falling or staying asleep” on a scale ranging from 0 (“none”) to 4 (“very severe”). Consistent with past research (8, 16, 32), we dichotomized the sleep item to indicate the presence of sleep problems: “present” (score ≥2 [i.e., moderate to very severe, indicating impact on daily function]) vs. “absent/mild” (score <2 [i.e., no impact on daily function]).

#### Post-concussive symptoms

The remaining items from the NSI were used to measure *post-concussive symptoms* (31). Participants rated a variety of vestibular (e.g., loss of balance), sensory (e.g., headaches), behavioral (e.g., feeling anxious), and cognitive (e.g., poor concentration) symptoms on a scale ranging from 0 (“none”) to 4 (“very severe”). The validity and reliability of the NSI, including in post-9/11 Veterans with mTBI, has been documented (7, 33).

#### Participation restrictions

The 8-item participation index of the Mayo-Portland Adaptability Inventory-4 (M2PI) was used to measure participation (34). Participants rate the following activities on a 5-point scale from 0 (i.e., no participation restriction) to 4 (i.e., severe participation restriction): initiation of activities; social contact; leisure and recreational activities; self-care; residence management (e.g., meal preparation); transportation; employment/other employment; and, financial management. The full Mayo-Portland Adaptability Inventory-4 is valid and reliable among community-based individuals living with TBI, and its subscales (i.e., the M2PI) can be used as standalone assessments (34–36). The M2PI has exhibited adequate psychometric properties when administered to post-9/11 Veterans with TBI (37).

#### Covariates

We included the following sociodemographic characteristics as covariates: *age* (in years at the time of the CTBIE); *gender* (male vs. female); *race* (Black, American Indian/Alaskan Native, Asian American, Native Hawaiian/Pacific Islander, White, other/unknown); *ethnicity* (Hispanic vs. non-Hispanic); *marital status* (married vs. non-married); *employment status* (unemployed, student/homemaker/volunteer, employed); and, pre-military *educational level* (high school or less vs. some college or more).

We included the following injury characteristics as covariates: *blast exposure* (yes vs. no); *pre-deployment TBI* (yes vs. no); *post-deployment TBI* (yes vs. no); and, presence of *any alteration of consciousness* (yes vs. no), *any loss of consciousness* (yes vs. no), or *any post-traumatic amnesia* (yes vs. no).

Lastly, we included indicators of co-morbid mental and medical conditions as covariates. We included indicators of co-morbid *posttraumatic stress disorder* (PTSD; yes vs. no) and *depression* (yes vs. no) using ICD-9 or −10 codes within the VHA electronic medical record documented between one year prior and 90 days following the CTBIE. Probable PTSD and depression was determined by the presence of corresponding ICD-9 or −10 codes associated with either (1) two outpatient encounters or (2) one inpatient encounter during the above timeframe. *Medical comorbidity* (yes vs. no) was measured using the Charlson comorbidity index, derived using ICD-9 or -10 codes documented between one year prior and 90 days following the CTBIE (38).

### Data analysis

We performed a descriptive analysis for observed and latent variables. Latent variable path analysis with a robust maximum likelihood estimator was used to evaluate hypotheses using Mplus Version 8.6 (39). For the measurement component of the model, consistent with default Mplus procedures, we identified latent variables of post-concussive symptoms and participation restrictions by estimating factor loadings and constraining latent variable means/intercepts to 0 and the factor variance to 1. The measurement (i.e., latent variables and covariance of residuals) and structural components of the model (i.e., inclusion of hypothesized paths amongst observed and latent variables) were evaluated and refined based on widely adopted model fit criteria (i.e., RMSEA ≈ 0.06; CFI ≈ 0.95; TLI ≈ 0.95; SRMR ≈ 0.08) (40) as well as theoretical rationale.

Upon arriving at a measurement model that fit the data reasonably well, we estimated the bivariate correlations between sleep problems and latent variables. We then specified two theoretically plausible latent variable path models. For *Model 1*, the following paths were estimated: (1) the path from sleep problems to latent indicators of post-concussive symptoms; and, (2) the path from each latent indicator of post-concussive symptoms to latent indicators of participation. For *Model 2*, we added the paths estimating the relationship between sleep problems and latent indicators of participation. We used BIC and a descriptive analysis of global fit indices (e.g., RMSEA) to compare model fit, thus determining which of the competing models was most consistent with the data.

Hypothesized mediation effects were tested using the product of coefficients method, from which we derived estimates of the indirect effect of sleep problems upon participation restrictions, as mediated by latent indicators of post-concussive symptoms (41). All estimates were adjusted for the aforementioned covariates. Statistical significance for all parameter estimates was evaluated at *α* = 0.05.

## Results

A substantial portion of Veterans in the sample reported sleep problems (87%). The average age in our sample was 36 years, and most Veterans were White (67%), male (93%), and non-Hispanic (84%). Most reported exposure to a blast (71%), with a notable portion of the sample experiencing co-morbid PTSD (48%) and depression (22%). See Table 1.

**Table 1:** Characteristics of Veterans with mTBI (*n* = 8,733).

Participant Characteristic		Sleep Problems	
	Total Sample	Absent, *n* = 1,175 (13.5%)	Present, *n* = 7,558 (86.5%)
	*n* (*%*)	*n* (*%*)	*n* (*%*)
Age, mean (SD)	36.10 (8.18)	35.59 (8.42)	36.18 (8.14)
Female (vs. male)	609 (7.0%)	67 (5.7%)	542 (7.2%)
Race			
Black	1,566 (17.9%)	158 (13.4%)	1,408 (18.6%)
White	5,866 (67.2%)	841 (71.6%)	5,025 (66.5%)
American Indian/Alaskan Native	101 (1.2%)	14 (1.2%)	87 (1.2%)
Asian American	238 (2.7%)	43 (3.7%)	195 (2.6%)
Native Hawaiian/Pacific Islander	207 (2.4%)	24 (2.0%)	183 (2.4%)
Other/unknown	755 (8.6%)	95 (8.1%)	660 (8.7%)
Hispanic ethnicity (vs. no)	1,441 (16.5%)	178 (15.1%)	1,263 (16.7%)
Married (vs. not married)	5,129 (58.7%)	712 (60.6%)	4,417 (58.4%)
Employment status			
Employed	4,260 (48.8%)	673 (57.3%)	3,587 (47.5%)
Student/homemaker/volunteer	1,623 (18.6%)	227 (19.3%)	1,396 (18.5%)
Unemployed	2,850 (32.6%)	275 (23.4%)	2,575 (34.1%)
Some college or more (vs. high school or less)	3,514 (40.2%)	474 (40.3%)	3,040 (40.2%)
Blast exposure (vs. no)	6,210 (71.1%)	787 (67.0%)	5,423 (71.8%)
Pre-deployment TBI (vs. no)	2,304 (26.4%)	338 (28.8%)	1,966 (26.0%)
Post-deployment TBI (vs. no)	1,525 (17.5%)	193 (16.4%)	1,332 (17.6%)
Alteration of consciousness (vs. no)	8,155 (93.4%)	1,095 (93.2%)	7,060 (93.4%)
Loss of consciousness (vs. no)	4,052 (46.4%)	486 (41.4%)	3,566 (47.2%)
Post-traumatic amnesia (vs. no)	2,973 (34.0%)	323 (27.5%)	2,650 (35.1%)
PTSD (vs. no)	4,208 (48.2%)	365 (31.1%)	3,843 (50.8%)
Depression (vs. no)	1,874 (21.5%)	170 (14.5%)	1,704 (22.5%)
Medical comorbidity (vs. no)	655 (7.5%)	71 (6.0%)	584 (7.7%)

mTBI, mild traumatic brain injury; TBI, traumatic brain injury; SD,standard deviation.

### Measurement component of the model

We first specified an unconditional measurement model, identifying three latent indicators of post-concussive symptoms and two latent indicators of participation (five total). All latent variable covariances were freely estimated. The model exhibited reasonable fit per standard global fit indices (RMSEA = 0.06, CFI = 0.92, TLI = 0.91, SRMR = 0.04).

The three latent indicators of post-concussive symptoms represented the following domains, similar to domains empirically derived in a prior study (7): *Vestibular-Sensory* (11 items; e.g., headache); *Mood-Behavioral* (5 items; e.g., frustration); and, *Cognitive* (4 items; e.g., forgetfulness). The hearing item was removed because it is consistent with prior examinations of the factor structure of the NSI and because it exhibited a relatively weak factor loading to the vestibular-sensory latent construct (0.50) (7, 42).

We identified two latent indicators of participation restrictions: (1) *Social and community participation* (comprised of initiation, social contact, and leisure items); and, (2) *Productivity* (comprised of self-care, residence management, transportation, and financial management items). We identified two distinct latent indicators of participation because the model with a single latent indicator of participation exhibited inadequate model fit (RMSEA = 0.17, CFI = 0.78, TLI = 0.67, SRMR = 0.08) (40). Further, theory holds that participation is multi-faceted, comprised of varying “types” of participation that can be distinguished based on the nature of activity engagement (e.g., social vs. productivity-based) (43). Consistent with prior studies (37, 44), the employment indicator was excluded due to low factor loadings on latent constructs (<0.40). See Table 2 for a summary of the measurement model.

**Table 2:** Characteristics of the measurement component of the model (*n* = 8,733).

Observed Indicator of Latent Constructs	Factor Loading (SE)	Mean (SD)
Vestibular-Sensory Symptoms		
Dizziness	0.72 (0.01)	1.35 (0.96)
Balance	0.74 (0.01)	1.30 (0.99)
Coordination	0.72 (0.01)	1.31 (1.03)
Headaches	0.59 (0.01)	2.38 (1.08)
Nausea	0.61 (0.01)	1.02 (1.07)
Vision	0.64 (0.01)	1.51 (1.12)
Light	0.61 (0.01)	1.88 (1.19)
Noise	0.62 (0.01)	1.77 (1.23)
Numbness	0.60 (0.01)	1.66 (1.25)
Taste/Smell	0.57 (0.01)	0.72 (1.05)
Appetite	0.62 (0.01)	1.33 (1.23)
Mood-Behavioral Symptoms		
Fatigue	0.68 (0.01)	2.20 (1.17)
Anxiety	0.79 (0.01)	2.57 (1.11)
Depression	0.76 (0.01)	2.02 (1.27)
Irritability	0.81 (0.01)	2.64 (1.10)
Frustration	0.86 (0.01)	2.36 (1.20)
Cognitive Symptoms		
Concentration	0.82 (0.01)	2.34 (1.09)
Forgetfulness	0.79 (0.01)	2.50 (1.05)
Decisions	0.82 (0.01)	1.72 (1.23)
Thinking	0.85 (0.01)	2.01 (1.19)
Social and Community Participation		
Initiation	0.69 (0.01)	1.96 (1.29)
Social contact with others	0.75 (0.01)	2.47 (1.29)
Leisure and recreational activities	0.77 (0.01)	2.11 (1.36)
Productivity		
Self-care	0.72 (0.01)	0.51 (0.89)
Residence management	0.85 (0.01)	0.70 (1.11)
Transportation	0.62 (0.01)	0.41 (0.89)
Financial management	0.66 (0.01)	0.86 (1.26)

Factor loadings are standardized; mTBI, mild traumatic brain injury; SE, standard error; SD, standard deviation.

### Structural component of the model

Examination of the bivariate correlations between sleep problems and latent indicators signaled relationships consistent with the hypothesized model. Specifically, those with sleep problems reported more severe post-concussive symptoms across domains, as well as greater restrictions in social and community participation and productivity. Similarly, more severe post-concussive symptoms were associated with greater restrictions in social and community participation and productivity. See Table 3.

**Table 3:** Bivariate correlations for key study variables (*n* = 8,733).

Variable	Bivariate Correlations					
	1	2	3	4	5	6
1. Sleep Problems^a^	—					
2. Vestibular-Sensory Symptoms	0.32	—				
3. Mood-Behavioral Symptoms	0.39	0.72	—			
4. Cognitive Symptoms	0.30	0.74	0.82	—		
5. Social / Community Participation	0.23	0.45	0.59	0.54	—	
6. Productivity	0.16	0.42	0.40	0.43	0.59	—

*p *< 0.001 for all estimates.

^a^ Values represent point-biserial correlations.

We first specified *Model 1*, estimating the following paths: (1) sleep problems to each latent indicator of post-concussive symptoms (vestibular-sensory; mood-behavioral; cognitive); and, (2) the path from each latent indicator of post-concussive symptoms to each latent indicator of participation (social and community participation; productivity). This model fit the data well (RMSEA = 0.04, CFI = 0.91, TLI = 0.89, SRMR = 0.03) (40).

**Figure 1:**
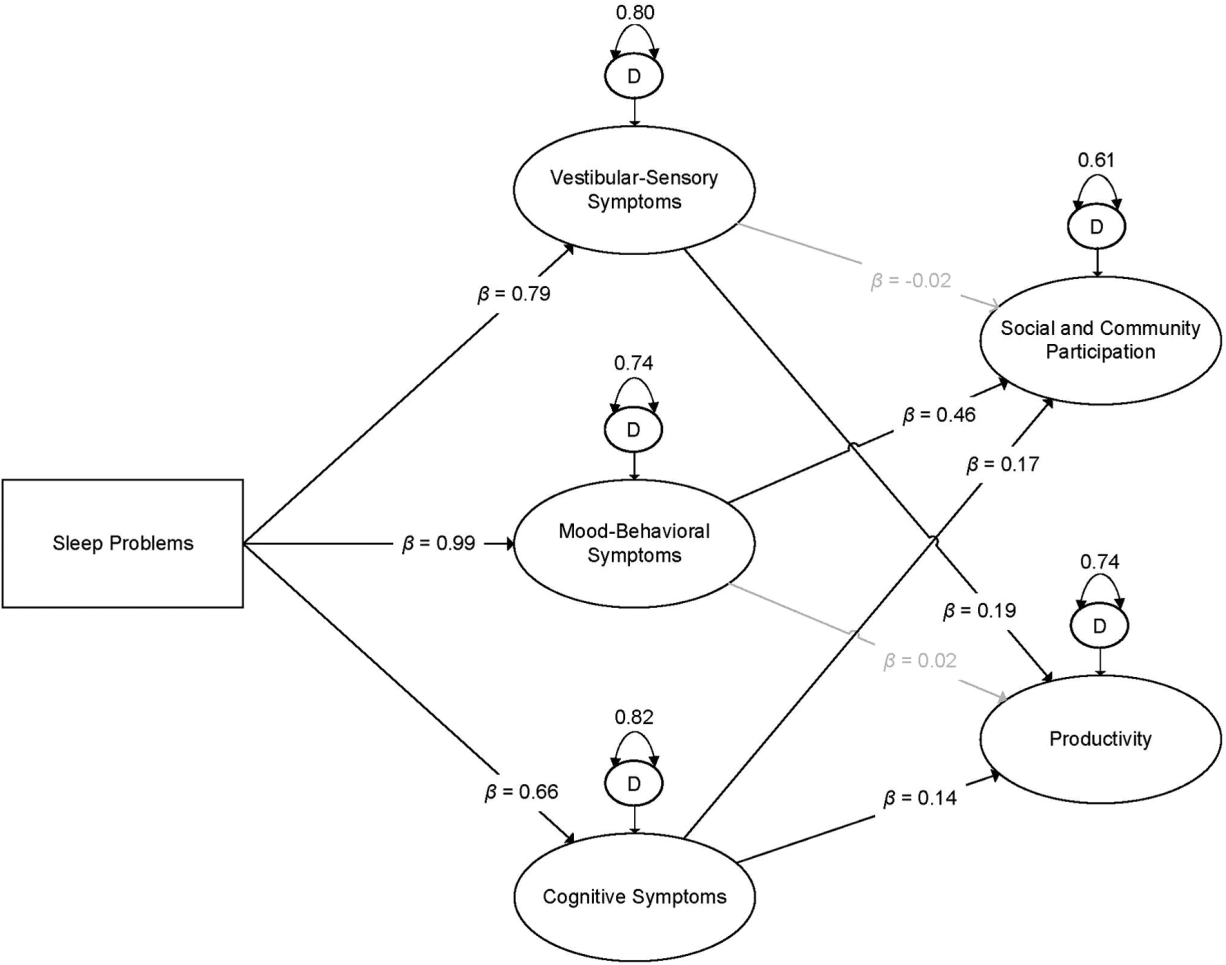
Visual illustration of the final latent variable path model. Boxes indicate observed variables; ovals indicate latent indicators. Dark lines indicate estimates that were statistically significant at *α* = 0.05; gray lines indicate relationships that were not statistically significant. *β *= standardized estimate; D = disturbance term (i.e., residual variance of latent indicator). All estimates are adjusted for sociodemographic, injury-related, and co-morbidity co-variates.

Subsequently, we specified *Model 2*, which included the additional paths from sleep problems to each latent indicator of participation, adjusted for post-concussive symptoms. We rejected Model 2 for two reasons. First, the added model complexity needed to specify Model 2 did not result in improved model fit compared to Model 1. Specifically, a descriptive analysis of model fit indices indicated identical model fit for Models 1 and 2 (RMSEA = 0.04, CFI = 0.91, TLI = 0.89, SRMR = 0.03). Further, examination of the BIC values for Model 1 (614066.81) and Model 2 (614077.38) yielded a BIC difference of 10.58, providing “very strong evidence” in favor of the more parsimonious model (Model 1) (45). Second, examination of the parameter estimates for the direct effects revealed the emergence of a negative suppressor effect (46). Specifically, while both theory and observed bivariate relations between sleep problems and productivity support a positive relationship (*r *= 0.16, *p* < 0.001)*,* when included in the model with the latent indicators of post-concussive symptoms, a statistically significant and *negative* relationship between sleep problems and productivity emerged (*b *= −0.05, *p* = 0.004). It is best practice to remove one or both variables causing the suppressor effect and retain the more parsimonious model, assuming that doing so is consistent with relevant theory (46). As such, we rejected Model 2 and interpreted Model 1. See Figure 1 for a visual illustration of Model 1.

#### Indirect effect of sleep problems upon social and community participation

The indirect effects of sleep problems upon restrictions in social and community participation offered mixed support for hypotheses. Sleep problems were associated with greater restrictions in social and community participation, as mediated by mood-behavioral and cognitive symptoms. However, there was no evidence that vestibular-sensory symptoms mediated the relationship between sleep problems and social and community participation. Sleep problems were associated with more severe vestibular-sensory, mood-behavioral, and cognitive post-concussive symptoms. In turn, more severe mood-behavioral and cognitive, but not vestibular-sensory symptoms, were associated with greater restrictions in social and community participation. See Table 4 for indirect effect estimates and Table 5 for specific parameter estimates.

**Table 4:** Parameter estimates for indirect effects (*n* = 8,733).

Indirect Effects	*b* (*SE*)	*β*	95% CI	*p*
Indirect Effects of Sleep Problems Upon Social and Community Participation				
Sleep problems → Vestibular-Sensory Symptoms → Social and Community Participation	−0.01 (0.01)	−0.01	−0.037, 0.017	0.476
Sleep problems → Mood-Behavioral Symptoms → Social and Community Participation	0.36 (0.02)	0.41	0.313, 0.408	<0.001
Sleep problems → Cognitive Symptoms → Social and Community Participation	0.11 (0.02)	0.13	0.076, 0.149	<0.001
Indirect Effects of Sleep Problems Upon Productivity				
Sleep problems → Vestibular-Sensory Symptoms → Productivity	0.10 (0.01)	0.16	0.078, 0.123	<0.001
Sleep problems → Mood-Behavioral Symptoms → Productivity	0.01 (0.02)	0.02	−0.016, 0.042	0.369
Sleep problems → Cognitive Symptoms → Productivity	0.09 (0.01)	0.14	0.069, 0.114	<0.001

*b*, unstandardized parameter estimate; *SE*, standard error; 95% CI, 95% confidence interval for standardized estimates; all estimates are adjusted for sociodemographic, injury-related, and co-morbidity covariates.

**Table 5:** Parameter estimates for final latent variable path model (*n* = 8,733).

Explanatory Variable	Dependent Variables									
	Vestibular-Sensory Symptoms		Mood-Behavioral Symptoms		Cognitive Symptoms		Social / Community Participation		Productivity	
	*b* (*SE*)	*β*	*b* (*SE*)	*β*	*b* (*SE*)	*β*	*b* (*SE*)	*β*	*b* (*SE*)	*β*
Sleep problems (vs. none)	0.54 (0.02)***	0.79	0.79 (0.03)***	0.99	0.66 (0.03)***	0.74	–	–	–	–
Post-concussive symptoms										
Vestibular-Sensory	–	–	–	–	–	–	−0.02 (0.03)	−0.01	0.19 (0.02)***	0.20
Mood-Behavioral	–	–	–	–	–	–	0.46 (0.03)***	0.41	0.02 (0.02)	0.02
Cognitive	–	–	–	–	–	–	0.17 (0.03)***	0.17	0.14 (0.02)***	0.19
Covariates										
Age	0.01 (0.00)***	−0.03	−0.01 (0.00)***	−0.05	−0.00 (0.00)*	−0.03	0.00 (0.00)**	0.03	−0.00 (0.00)*	−0.02
Female (vs. male)	0.19 (0.03)***	0.28	0.14 (0.03)***	0.18	0.12 (0.04)**	0.13	.02 (0.04)	0.02	−0.09 (0.03)**	−0.14
Race (vs. White)										
Black	0.15 (0.02)***	0.22	0.07 (0.02)**	0.08	0.01 (0.03)	0.01	−0.00 (0.03)	−0.01	−0.02 (0.02)	−0.03
American Indian/Alaskan Native	0.15 (0.07)*	0.22	−0.05 (0.07)	−0.06	0.06 (0.09)	0.07	0.03 (0.08)	0.04	0.07 (0.07)	0.11
Asian American	−0.05 (0.04)	−0.07	−0.14 (0.05)**	−0.17	−0.15 (0.05)**	−0.17	0.08 (0.06)	0.09	0.05 (0.04)	0.07
Native Hawaiian/Pacific Islander	0.10 (0.05)	0.14	0.00 (0.00)	0.00	0.01 (0.06)	0.01	0.18 (0.06)**	0.21	0.05 (0.05)	0.07
Other/unknown	0.06 (0.03)*	0.09	−0.01 (0.03)	−0.01	−0.00 (0.03)	−0.00	0.05 (0.04)	0.06	0.01 (0.03)	0.01
Hispanic ethnicity (vs. no)	0.07 (0.02)**	0.10	0.04 (0.02)	0.05	−0.02 (0.03)	−0.02	0.00 (0.03)	0.00	0.04 (0.02)*	0.06
Married (vs. not married)	0.06 (0.02)***	0.09	0.07 (0.02)***	0.09	0.06 (0.02)**	0.07	−0.08 (0.02)***	−0.09	0.08 (0.01)***	0.13
Employment status (vs. employed)										
Student/homemaker/ volunteer	−0.06 (0.02)**	−0.08	−0.04 (0.02)	−0.05	0.01 (0.03)	0.01	0.07 (0.03)*	0.08	0.10 (0.02)***	0.16
Unemployed	0.13 (0.02)***	0.19	0.16 (0.02)***	0.20	0.20 (0.02)***	0.23	0.13 (0.02)***	0.14	0.24 (0.02)***	0.37
Some college or more (vs. high school or less)	0.03 (0.02)*	0.05	−0.01 (0.02)	−0.01	0.01 (0.02)	0.01	0.01 (0.02)	0.01	0.01 (0.01)	0.01
Blast exposure (vs. no)	0.06 (0.02)***	0.09	0.08 (0.02)***	0.11	0.08 (0.02)***	0.09	−0.00 (0.02)	−0.00	0.01 (0.02)	0.01
Pre-deployment TBI (vs. no)	0.00 (0.02)	0.00	−0.01 (0.02)	−0.01	−0.00 (0.02)	−0.00	−0.08 (0.02)***	−0.09	−0.04 (0.02)**	−0.06
Post-deployment TBI (vs. no)	0.13 (0.02)***	0.19	0.11 (0.02)***	0.14	0.16 (0.03)***	0.18	−0.03 (0.02)	−0.03	0.04 (0.02)*	0.06
Alteration of consciousness (vs. no)	0.11 (0.03)***	0.17	0.07 (0.04)*	0.09	0.08 (0.04)	0.09	−0.02 (0.04)	−0.03	−0.04 (0.03)	−0.06
Loss of consciousness (vs. no)	0.09 (0.02)***	0.14	0.04 (0.02)*	0.05	0.07 (0.02)***	0.08	0.07 (0.02)**	0.07	0.07 (0.01)***	0.11
Post-traumatic amnesia (vs. no)	0.12 (0.02)***	0.17	0.10 (0.02)***	0.12	0.17 (0.02)***	0.19	−0.01 (0.02)	−0.01	0.00 (0.02)	0.00
PTSD (vs. no)	0.16 (0.02)***	0.23	0.31 (0.02)***	0.39	0.29 (0.02)***	0.33	0.16 (0.02)***	0.19	0.14 (0.02)***	0.22
Depression (vs. no)	0.17 (0.02)***	0.25	0.32 (0.02)***	0.40	0.27 (0.02)***	0.30	0.18 (0.02)***	0.21	0.12 (0.02)***	0.18
Medical comorbidity (vs. no)	0.04 (0.03)	0.06	−0.00 (0.03)	−0.00	0.01 (0.04)	0.01	−0.02 (0.03)	−0.02	0.02 (0.03)	0.03

*b*, unstandardized parameter estimate; *SE*, standard error; *β*, standardized parameter estimate; TBI, traumatic brain injury; * *p *< 0.05, ** *p *< 0.01, *** *p *< 0.001.

#### Indirect effect of sleep problems upon productivity

Similarly, we identified mixed support for our hypotheses regarding the indirect effect of sleep problems upon productivity. Sleep problems were associated with greater productivity restrictions, as mediated by vestibular-sensory and cognitive symptoms. There was no evidence that mood-behavioral symptoms mediated the relationship between sleep problems and productivity restrictions. As previously stated, sleep problems were associated with more severe post-concussive symptoms across all domains. More severe vestibular-sensory and cognitive symptoms, but not mood-behavioral symptoms, were associated with greater productivity restrictions.

## Discussion

In a national sample of 8,733 Veterans with clinician-confirmed mTBI, we examined whether post-concussive symptoms mediated the relationship between sleep problems and participation restrictions. Generally, our proposed model was supported, but the indirect effect of sleep problems differed when considering distinct types of participation. While sleep problems were associated with more severe post-concussive symptoms across all three domains (vestibular-sensory, mood-behavioral, and cognitive), the impact of such symptoms upon Veterans' participation depended on the specific nature of the activities (social and community participation vs. productivity). Findings underscore the importance of integrating evidence-based sleep treatment in the rehabilitative care of Veterans with mTBI. Further, our findings can inform efforts aimed at tailoring such care to their individualized clinical needs.

Study findings provide empirical support for evidence-based sleep treatment occupying a prominent role in the rehabilitation of Veterans with mTBI. It has been recommended that post-concussive sleep problems be prioritized in those with mTBI because they are amenable to treatment and their reduction contributes to overall post-concussive recovery (18–20, 47). Our findings bolster and extend these claims by offering evidence of relationships which suggest that by prioritizing the treatment of sleep problems, rehabilitation services may also ameliorate participation restrictions in Veterans with mTBI. For instance, providing cognitive behavioral therapy for insomnia (CBT-I), an evidence-based treatment for insomnia, has been linked to both reduced neurobehavioral symptoms (e.g., depression) and enhanced participation in Veterans (48, 49). Enhancing Veterans' participation is an organizing principle of VHA rehabilitative care (28, 50). Our findings indicate that VHA rehabilitative care could achieve this worthy objective by prioritizing the clinical management of sleep problems.

Systematic effort should be devoted to aligning “real-world” rehabilitation for post-concussive sleep problems with evidence-based recommendations. First, we echo previous calls for the screening of sleep problems for individuals with TBI, including in rehabilitation settings (51, 52). Systematically detecting sleep problems is requisite for subsequent clinical management. Second, according to the VA/DoD Clinical Practice Guideline (CPG) for mTBI (29), clinical management of identified post-concussive sleep problems should align with recommendations within the VA/DoD CPG for the management of insomnia and obstructive sleep apnea (OSA; Sleep CPG) (53). The Sleep CPG includes evidence-based recommendations for the assessment and treatment of insomnia and OSA, two sleep conditions for which Veterans with mTBI are at disproportionately high risk (54). However, adherence to such recommendations within clinical practice may be variable. For example, there are documented barriers to the delivery of guideline-recommended care for post-concussive sleep problems in VHA rehabilitation settings (e.g., provider awareness) (55). Further, Veterans with mTBI and associated participation restrictions may experience unique barriers to accessing specialized sleep treatment. For example, sequalae of mTBI (e.g., cognitive impairment) may exacerbate logistical challenges (e.g., transportation) that pose a barrier to accessing sleep treatment among those without mTBI (56). Strategies that systematically target barriers to Veterans' receipt of evidence-based treatment for post-concussive sleep problems should be developed and evaluated (57). By enhancing the quality of care received, such efforts could promote sleep quality and overall post-concussive recovery among Veterans with mTBI.

Cognitive symptoms were the only post-concussive symptom domain observed to mediate the relationship between sleep problems and both indicators of participation, suggesting a potentially broad impact upon Veterans' functioning following mTBI. This finding is consistent with a recent study in a sample of Veterans with mTBI that found participation restrictions were primarily associated with cognitive symptoms, but not other post-concussive symptom domains (9). Our study expands upon these findings by providing evidence that such cognitive symptoms may be downstream from sleep problems, expanding potential treatment targets for rehabilitative care aiming to promote Veterans' participation. Preliminary evidence indicates that sleep treatments may enhance cognitive function in those with TBI (58–60), although additional scientific investment is needed (52). Further, according to the VA/DoD mTBI CPG (29), Veterans with post-concussive cognitive impairment should receive specialized cognitive rehabilitation services (e.g., compensatory cognitive training) due to documented benefits on cognitive function, including among post-9/11 Veterans with mTBI (61).

By examining two distinct indicators of participation, we revealed divergent impacts of vestibular-sensory and mood-behavioral symptoms upon Veterans' functioning. Our study extends prior work which adopted an overall summary score of participation, observing that post-concussive symptoms across domains undermined Veterans' participation (8). Our findings suggest that such an approach may obscure more nuanced relationships between post-concussive symptoms and Veteran participation challenges, an understanding of which can enhance rehabilitative care for those with mTBI. For example, in our study, mood-behavioral symptoms were associated with restrictions in social and community participation (e.g., social contact), but not productivity (e.g., self-care). This may indicate that the interpersonal aspect of social and community participation may be challenging for those with chiefly mood-behavioral post-concussive impairments. Indeed, Veterans with such challenges (e.g., depression) report difficulty with securing healthy social bonds with others (62, 63). These interpersonal challenges may not necessarily translate to participation in productivity-related activities, the performance of which may occur in isolation from others. Adopting such a nuanced perspective on the link between post-concussive symptoms and participation challenges among Veterans with mTBI can inform priority targets of intervention according to clinical presentation, enabling more individualized rehabilitative care.

Studies examining participation in Veterans with mTBI, including the present study, typically emphasize the observable aspects of participation (e.g., independence), overlooking the subjective dimension of this complex construct (1, 64). Future research that examines the inter-relationships between sleep problems, post-concussive symptoms, and participation restrictions should also consider the meaning associated with Veterans' daily participation, or the extent to which daily activity aligns with their values and interests (1, 65). Engagement in meaningful activity is a critical ingredient for Veterans' community reintegration and contributes to their overall wellbeing, making it an important target for the rehabilitation of Veterans with mTBI (66, 67). Further, measuring all dimensions of participation may further elucidate the impact of treating sleep problems and other post-concussive symptoms on the daily lives of Veterans. For example, a recent study evaluating the efficacy of CBT-I in Veterans found improvements in the meaningfulness of daily activity, but not in the observable aspects of participation (i.e., performance) (49). Advancing understanding of such relationships could enable the refinement of rehabilitative care to better meet the individualized needs of Veterans with post-concussive sleep problems.

### Study limitations

The cross-sectional nature of this study precludes definitive conclusions regarding the temporal order of observed relationships. We could not identify whether sleep problems contribute to other post-concussive symptoms, or whether the reverse is more consistent with the data. However, the implied temporal order of our proposed model is supported by substantial theoretical and empirical support indicating that improved sleep quality is associated with enhanced overall post-concussive recovery (18–20, 47–49, 52, 68). Nonetheless, longitudinal studies should be conducted to disentangle the temporal ordering of sleep problems, other post-concussive symptoms, and participation restrictions. Further, many Veterans who received the CTBIE did not receive the M2PI, and the extent to which Veterans' receipt of the M2PI systematically varied is unclear. Such systematic variation may threaten the generalizability of findings. In addition, our findings were collected in a sample of Veterans receiving outpatient VHA care, and findings may not generalize to other Veterans or to civilian populations. However, the generalizability of our findings benefits from the national sample of Veterans included in this study. Nonetheless, findings should be replicated in other populations (e.g., civilians with sports-related concussion). In the current study, we did not account for symptom validity using embedded validity scales (e.g., Validity-10) (69) for two primary reasons. First, prior examinations of self-reported NSI scores among Veterans indicate that removing individuals based on embedded validity scales may undermine measurement precision among those with more severe symptoms (7). Second, invalid responding is a complex behavior, the identification of which requires the integration of multiple sources of data (e.g., Mild Brain Injury Atypical Symptoms scale) (70) rather than the use of embedded validity scales in isolation (7, 71). Future research should replicate study results among valid responders identified using multiple sources of data that were unavailable for the current study. Sleep problems were measured using a single self-reported indicator, and future research should attempt to replicate findings using standardized measures of sleep problems (e.g., Pittsburgh Sleep Quality Index) (72). Further, the effects of clinical conditions underlying such sleep problems (e.g., insomnia) upon observed relationships should also be studied. Finally, we were unable to account for all potentially confounding variables in our model. For example, we were unable to adjust for the influence of environmental factors, which are inextricably connected to participation (73). However, we accounted for many theoretically plausible confounding variables (e.g., co-morbid PTSD and depression). Nonetheless, future studies should expand upon the set of covariates used in this study.

## Conclusion

In this study, we found that post-concussive symptoms mediated the relationship between sleep problems and participation restrictions in Veterans with mTBI, although the indirect effect of sleep problems varied across different types of participation. Sleep problems were associated with more severe post-concussive symptoms across all domains. However, effects of post-concussive symptoms were conditional on the specific nature of participation (i.e., social and community participation vs. productivity). Findings underscore the importance of integrating evidence-based sleep treatment in the rehabilitative care of Veterans with mTBI and can inform efforts aimed at tailoring rehabilitative care to their individualized needs.

## Data Availability

The data analyzed in this study is subject to the following licenses/restrictions: N/A. Requests to access these datasets should be directed to adam.kinney@va.gov.
